# Machine learning for early prediction of preterm birth

**DOI:** 10.1186/s12884-026-09784-w

**Published:** 2026-08-10

**Authors:** Firanol Teshome, Netsanet Workneh Gidi, Se-woon Choe, Jude Kong, Gelan Ayana

**Affiliations:** 1 School of Biomedical Engineering, Jimma Institute of Technology, Jimma University, 378 Jimma, Ethiopia; 2 Department of Pediatrics & Child Health, Jimma Institute of Health, Jimma University, 378 Jimma, Ethiopia; 3 Department of Biomedical Engineering, Kumoh National Institute of Technology, Gumi, 39253 Republic of Korea; 4 Artificial Intelligence & Mathematical Modeling Lab (AIMMLab), Dalla Lana School of Public Health, University of Toronto, 155 College St, Toronto, ON M5T 3M7 Canada; 5 Department of Mathematics, Bahen Centre for Information Technology, University of Toronto, 40 St. George St, Toronto, ON M5S 2E4 Canada; 6 Africa-Canada Artificial Intelligence and Data Innovation Consortium (ACADIC), Toronto, Canada; 7 Global South Artificial Intelligence for Pandemic and Epidemic Preparedness and Response Network (AI4PEP), Toronto, Canada

**Keywords:** Preterm birth, Early prediction, Machine learning, Obstetric care, Risk stratification

## Abstract

**Background:**

Preterm birth (PTB), defined as delivery before 37 completed weeks of gestation, remains a major cause of neonatal mortality and long-term morbidity worldwide. Conventional risk assessment strategies, including cervical length measurement and biomarker-based screening, have shown limited predictive performance. Machine learning (ML) may improve PTB prediction by integrating heterogeneous clinical data and identifying complex risk patterns.

**Methods:**

A structured narrative review was conducted by systematically searching PubMed, Web of Science, Scopus, IEEE Xplore, and Google Scholar for studies published between January 2010 and April 2024. Studies were screened according to predefined eligibility criteria, and 14 studies evaluating ML models for PTB prediction using human pregnancy datasets were included in the final narrative synthesis. Data on study characteristics, predictor variables, ML methods, validation strategies, and model performance were extracted and synthesized narratively.

**Results:**

A total of 14 studies met the predefined eligibility criteria and were included in the final narrative synthesis. ML models generally performed better when they used longitudinal electronic health records (EHRs), repeated measurements, or richer maternal clinical histories. Ensemble approaches such as random forest, gradient boosting, and stacking models, as well as deep learning (DL) methods including artificial neural networks (ANNs), recurrent neural networks (RNNs), and long short-term memory networks (LSTMs), were frequently among the best-performing models. Across the included studies, previous PTB, cervical length, maternal age, hypertensive disorders, diabetes, and body mass index (BMI) emerged as the most consistently reported predictors of ML model performance. However, the evidence was highly heterogeneous in data sources, outcome definitions, validation methods, and performance reporting. External validation, calibration assessment, and real-world clinical evaluation were limited.

**Conclusion:**

ML shows considerable promise for improving early PTB risk stratification, but clinical translation will depend on early prediction, interpretable outputs, and robust external validation. Although ML models generally demonstrated promising predictive performance, interpretation of these findings is limited by methodological heterogeneity and inconsistent external validation across studies. Future studies should prioritize standardized reporting, multicenter external validation, calibration assessment, explainability, and prospective implementation studies to facilitate routine clinical adoption.

## Introduction

PTB, defined as delivery before 37 completed weeks of gestation, remains one of the leading causes of neonatal mortality and long-term morbidity worldwide [1, 2]. Despite advances in neonatal and obstetric care, PTB continues to pose a major public health challenge, contributing significantly to infant mortality, neurodevelopmental impairment, chronic health complications, and healthcare expenditures across both developed and developing countries [3–5]. The burden is particularly high in low-resource settings, where limited access to specialized maternal and neonatal care further increases the risk of adverse outcomes [6, 7]. PTB may occur spontaneously or result from medically indicated delivery due to maternal or fetal complications; however, regardless of etiology, one of the greatest challenges in obstetric care remains the timely identification of pregnancies at increased risk before irreversible pathological processes develop or opportunities for prevention are lost [8]. The pathogenesis of PTB is complex and multifactorial, involving interactions among maternal demographic characteristics, obstetric history, medical comorbidities, genetic predisposition, environmental exposures, and behavioral factors [4, 7, 9, 10]. This complexity has made accurate risk prediction particularly difficult using conventional clinical approaches. Current PTB risk assessment strategies include cervical length measurement, fetal fibronectin testing, biomarker-based screening, and clinical risk-factor assessment [11, 12]. Although these approaches have improved obstetric management, their predictive performance remains limited, particularly when applied to asymptomatic women or during early pregnancy [13]. Many traditional methods also evaluate risk factors individually or assume linear relationships between predictors and outcomes, which may limit their ability to capture the complex, nonlinear biological and clinical interactions underlying PTB [14, 15]. In this context, ML has emerged as a promising approach for integrating heterogeneous clinical data, identifying complex associations, and generating individualized risk estimates from routinely collected information [16–18].

**Fig. 1:**
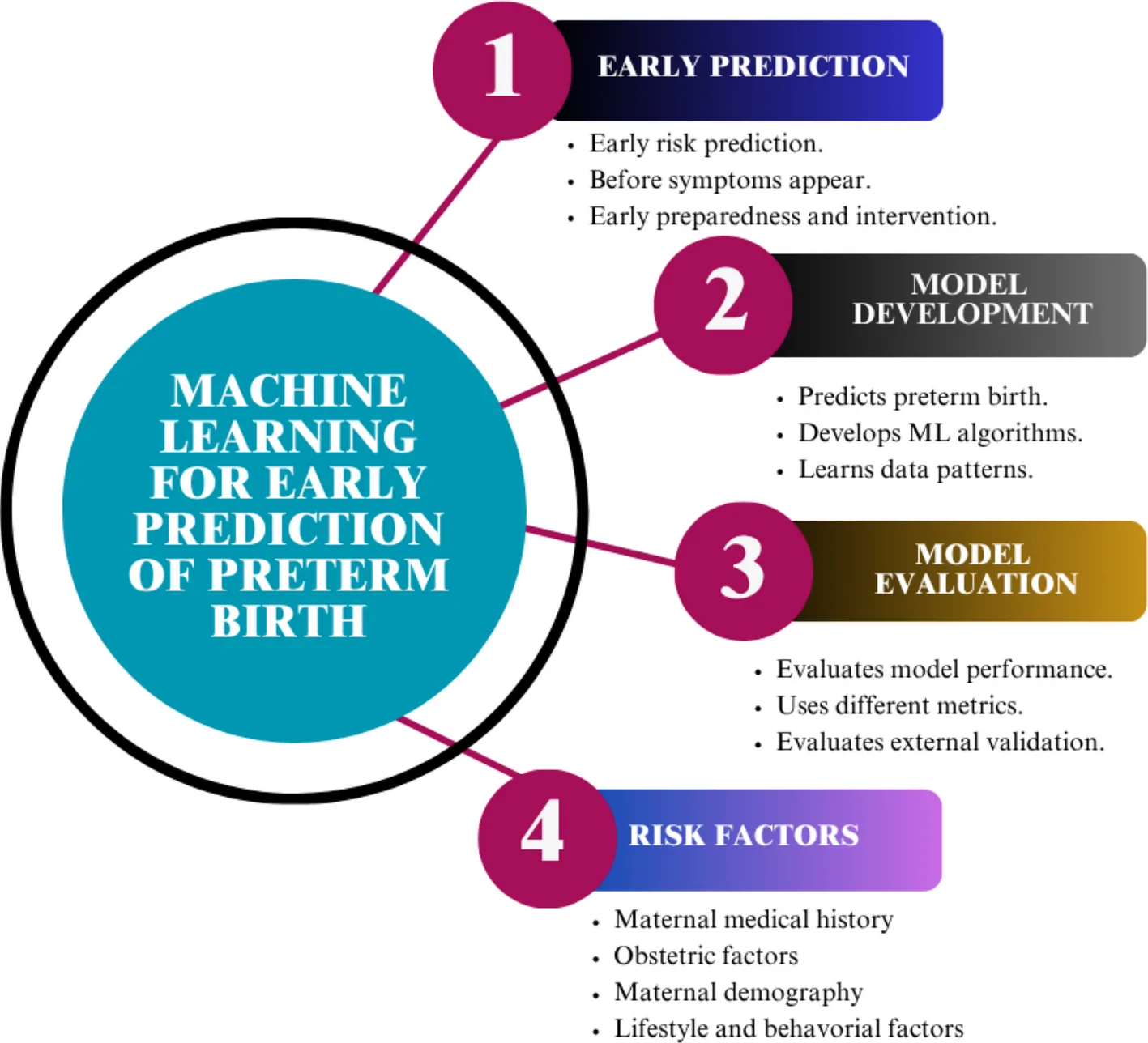
General overview of ML for early prediction of PTB

From a clinical perspective, the value of PTB prediction extends beyond identifying women at risk; it lies in identifying pregnancies for which meaningful action can be taken. Some predictors represent potentially modifiable risk factors, including BMI, ANC attendance, diabetes, hypertension, smoking, stress, and other clinical and lifestyle-related exposures. These factors may support prevention-oriented interventions and risk-reduction strategies. In contrast, predictors such as previous PTB, cervical length, multiple gestation, and other obstetric characteristics are largely non-modifiable but may identify women who would benefit from intensified surveillance, progesterone therapy, cervical cerclage, specialist referral, or more frequent prenatal monitoring [19]. Consequently, the clinical usefulness of any prediction model depends not only on predictive accuracy but also on its ability to identify actionable risk sufficiently early to influence clinical management. Figure 1 shows a general overview of ML application scope for early prediction of PTB.

Despite growing interest in ML-based PTB prediction, the literature remains heterogeneous in terms of data sources, feature selection strategies, model architectures, validation approaches, and performance reporting. Consequently, the relative clinical utility and translational readiness of existing models remain unclear. This narrative review synthesizes current evidence on ML approaches for PTB prediction, with emphasis on data characteristics, predictor importance, model performance, interpretability, and clinical applicability. Beyond summarizing existing evidence, this review identifies common methodological patterns, implementation challenges, and priorities for future ML research to facilitate translation into routine obstetric practice.

## Methods

This review was conducted as a structured narrative review of the literature on ML for the early prediction of PTB. The objective was to synthesize evidence on model performance, predictive variables, and clinical interpretability, with emphasis on studies that developed or evaluated ML-based prediction models for PTB in human pregnancy datasets.

### Search strategy

A literature search was performed in PubMed, Web of Science, Scopus, and Google Scholar for studies published between January 2010 and April 2024. The search strategy combined controlled vocabulary and free-text terms related to PTB and ML, including combinations of “preterm birth,” “preterm delivery,” “premature birth,” “machine learning,” “artificial intelligence,” “predictive model,” “risk prediction,” “deep learning,” “random forest,” “gradient boosting,” “XGBoost,” “neural network,” and “explainable AI.” Boolean operators (AND, OR) were used to combine the search terms. Reference lists of eligible articles and relevant reviews were also screened to identify additional studies.

### Eligibility criteria

Studies were considered eligible if they met all of the following criteria: (i) investigated the prediction of PTB in pregnant women or maternal cohorts; (ii) employed one or more ML-based data-driven predictive approaches; (iii) reported model development, validation, or comparative performance; and (iv) provided sufficient information on outcome definitions, predictor variables, and model evaluation metrics. Studies were excluded if they did not focus on PTB prediction, did not use ML methods, were editorials, commentaries, conference abstracts, duplicate publications, or did not provide sufficient extractable information relevant to the review objectives. Only studies published in English were included.

### Study selection and data extraction

**Fig. 2:**
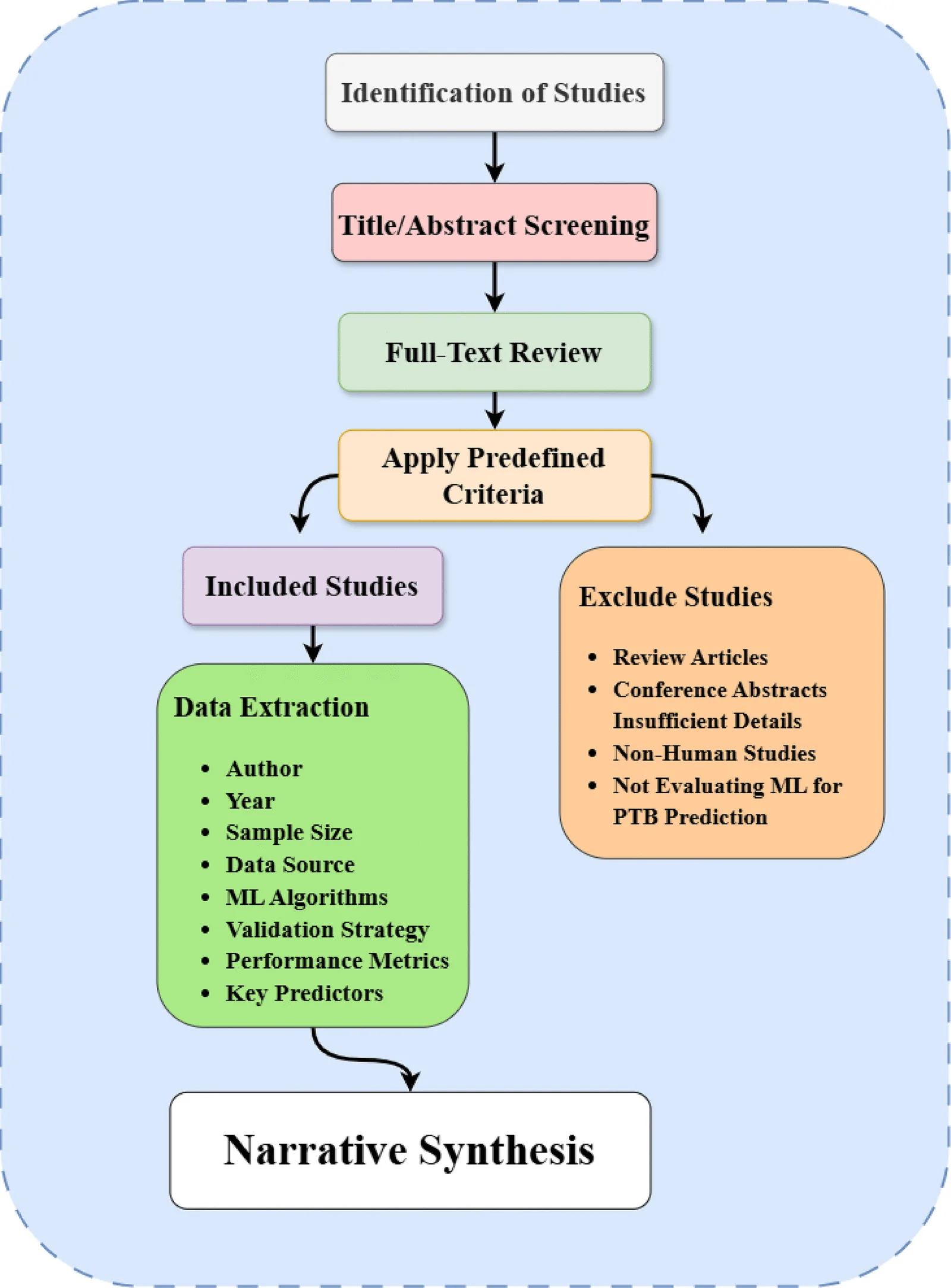
Methodology flow chart

Study selection involved title/abstract screening followed by full-text review using predefined eligibility criteria. Studies were included if they developed or evaluated ML models for PTB prediction using human pregnancy datasets and reported adequate methodological and performance data. Review articles, conference abstracts lacking sufficient methodological detail, non-human studies, and studies not evaluating ML for PTB prediction were excluded. 14 studies met the eligibility criteria and were included in the narrative synthesis. Data extracted included the first author, publication year, cohort characteristics, sample size, data source, ML algorithms, validation strategy, performance metrics, and key predictors. The study selection process is summarized in Fig. 2.

Data were extracted from each included study using a predefined data extraction form. The extracted information included general information like publication year, country, study design, data source, sample size, participant characteristics, predictor variables, ML algorithms, feature selection methods, validation strategy, performance metrics, and the main findings. The extracted data were used to compare study characteristics and summarize the evidence across studies.

### Data synthesis

Because the available evidence used heterogeneous outcomes, study designs, datasets, and evaluation metrics, quantitative meta-analysis was not considered appropriate. Findings were therefore synthesized narratively. Performance was interpreted in the context of outcome prevalence, validation strategy, and clinical usability. Where studies reported different metrics, direct numerical comparison was avoided unless the metrics were methodologically comparable. In addition to discrimination metrics such as AUC, accuracy, sensitivity, specificity, and root mean square error (RMSE) (for regression-based models). The review also considered the clinical relevance of predictors by distinguishing between modifiable factors and non-modifiable or less modifiable indicators. This framework was used to interpret how individual models may support prevention, surveillance, or early intervention.

A meta-analysis was not performed because of high heterogeneity in study populations, PTB definitions, predictor variables, ML methods, validation strategies, and reported performance metrics. Instead, the findings were synthesized narratively by comparing study characteristics, modeling approaches, and predictive performance to identify common patterns and evidence gaps.

## Results

### Common ML algorithms used in the early prediction of PTB

ML, a subset of artificial intelligence (AI), enables computers to learn from data and make predictions or decisions, uncovering patterns in complex datasets [20].

Several ML algorithms have been applied to PTB prediction to improve early risk identification beyond conventional approaches [16, 21, 22]. The reviewed studies included supervised learning methods such as logistic regression, decision trees, support vector machines (SVM), random forest, gradient boosting, and naive Bayes. Logistic regression was frequently used as an interpretable baseline [20, 23]. Decision trees have also been used to derive straightforward clinical decision rules from maternal demographics, obstetric history, and pregnancy-related factors [24]. Their transparent structure supports interpretability, although individual trees are often sensitive to data variation and may perform worse than ensemble methods [25, 26]. SVMs were applied to labeled classification tasks and were often combined with feature-selection methods to identify informative maternal and pregnancy-related variables [27]. Random forest models combine multiple trees to improve predictive stability, reduce overfitting, and handle heterogeneous clinical datasets [28]. Gradient-boosting methods, including XGBoost and LightGBM, were also frequently evaluated [29–32]. Naive Bayes classifiers were included as computationally efficient baseline models [33, 34]. DL approaches, including artificial neural networks (ANNs), recurrent neural networks (RNNs), and long short-term memory (LSTM) networks, were used in studies involving longitudinal or sequential pregnancy data [29, 35]. Ensemble and hybrid approaches were also reported [36].

### Study-level findings and reported performance

Across the reviewed studies, predictive performance varied according to cohort, timing of prediction, feature set, and outcome definition. In an early singleton-pregnancy dataset, Andrade Junior et al. [21] developed a stacking-based ensemble model that outperformed cervical length measurement alone, achieving an AUC of 0.808 compared with 0.3818 for the comparator. Rocha et al. [36] analyzed large maternal datasets from Brazilian municipalities and evaluated six ML algorithms, including XGBoost, elastic net, quantile ordinal regression (LASSO), linear regression, ridge regression, and decision tree. Model performance was assessed using root mean square error (RMSE), and XGBoost achieved the lowest RMSE, followed by linear regression, ridge regression, elastic net, LASSO, and decision tree in descending order for predicting delivery time. The main variables influencing delivery time included previous cesarean delivery, low ANC frequency, and maternal age.

Longitudinal and sequence-aware models were evaluated in settings where risk changed over time. Gao et al. [37] applied RNNs to EHRs from 25,689 deliveries and reported an AUC of 0.827 for extreme PTB prediction before 28 weeks. The most important predictors were multiple gestation, short cervical length, and maternal health complications. Similarly, Ebrahimvandi et al. [38] assessed logistic regression, random forest, and gradient boosting on large maternal data derived from U.S. birth records. Gradient boosting performed best, with an AUC of 0.75, sensitivity of 0.64, and specificity of 0.73 on validation data. The most important predictors in this study were gestational hypertension, interbirth interval, and history of PTB. Taken together, these studies indicate that ML models incorporating longitudinal EHRs and repeated clinical observations consistently achieved stronger predictive performance than models relying on single time-point assessments. Although RNNs and gradient boosting algorithms performed well, the findings suggest that access to temporally rich clinical data and clinically relevant predictors, including multiple gestation, cervical length, hypertensive disorders, and previous PTB, contributed more to predictive accuracy than algorithm choice alone.

Model performance also varied by parity and pregnancy stage. In nulliparous women, Sari et al. [34] found that ANN performance improved when first- and second-trimester data were combined, reaching an AUC of 0.8 after lower trimester-specific performance. In the first trimester, ANN showed an AUC of 0.60 on the validation set, and in the second trimester, performance improved slightly to 0.65. After combining data from both trimesters, predictive accuracy increased. The key predictors contributing to this model were diabetes mellitus, history of abortion, and pregnancy abnormalities. Overall, these findings demonstrate that predictive performance improves as additional clinical information becomes available throughout pregnancy. The observed increase in model performance after combining first- and second-trimester data suggests that integrating longitudinal maternal information enhances risk stratification, while also highlighting the importance of selecting prediction windows that allow sufficient time for preventive clinical interventions.

In a prospective cohort of nulliparous and multiparous women, Khan et al. [39] reported that XGBoost achieved AUC values of 0.735 in parous women and 0.723 in nulliparous women, and that the most important predictors differed by subgroup. In parous women, previous PTB, previous cesarean section, and preeclampsia were dominant predictors; in nulliparous women, BMI, maternal age, and amniotic infection were more prominent. Khabiti et al. [40] used a large dataset to classify term birth, spontaneous PTB, and provider-initiated PTB, and the combined decision tree, SVM, and random forest model achieved an accuracy of 0.81 and an AUC of 0.68. Wong et al. [41] applied logistic regression, decision trees, random forests, XGBoost, and MLPs in a population-based cohort and showed that the MLP achieved a classification accuracy of 0.57, while the overall comparison highlighted substantial variability across model classes. Raja et al. [42] also reported that SVM achieved an accuracy of 0.909 after entropy-based feature selection in a rural Indian cohort. In another study, Zhang et al. [43] applied LSTM to sequences of electronic medical record (EMR) data from early pregnancy to 28 weeks of gestation. LSTM achieved an accuracy of 0.739 and an AUC of 0.651. The major predictors identified in that study were blood pressure, blood glucose, and lipids. Abraham et al. [44] used dense EHR phenotyping and a gradient-boosted decision tree model with gestational age estimates at 0, 13, 28, and 35 weeks. The model achieved precision and recall of 0.96, although performance against manual chart review was substantially lower in AUC terms. Overall, these studies demonstrate that ML performance varied considerably across clinical populations, prediction tasks, and validation approaches. While several algorithms achieved promising discrimination or classification accuracy, differences in study design, outcome definitions, predictor availability, and evaluation metrics make direct comparison between models difficult. Collectively, the evidence suggests that model performance depends more on the quality and characteristics of the underlying data than on any single ML algorithm.

More recent studies further reported the use of large-scale data and advanced modeling approaches. Kong et al. [45] applied Automated Machine Learning (AutoML) frameworks to 715,962 inpatient discharge records and obtained median AUCs of 0.846–0.820, with XGBoost, stacked ensembles, and random forest ranking best. Qian et al. [46] built classical ML- and LSTM-based models from EHRs in a Chinese tertiary hospital, reporting AUCs of 0.826 for random forest and 0.851 for LSTM, with sustained performance above 0.80 in external validation. Liu et al. [47] developed an ML-guided nomogram for PTB risk from 2.57 million U.S. National Vital Statistics System (NVSS) records in the 2022 cohort and 2.69 million records for external validation. Using age, race, education, parity, interpregnancy interval, smoking, and prenatal care timing across four learners, the study achieved AUCs of 0.701–0.704 with good calibration and positive net benefit on decision-curve analysis.

Across the reviewed literature, commonly used data sources included large hospital EHRs, national birth registries such as the NVSS, and administrative inpatient databases, whereas EHG and microbiome datasets were smaller and often multi-cohort. Recurring predictive features included prior PTB, multiple gestation, premature rupture of membranes (PROM), preeclampsia, incompetent cervix, maternal age, parity, BMI, and socioeconomic proxies such as education and insurance. In contrast, EHG and microbiome models emphasized temporal and nonlinear descriptors such as entropy, spectral features, and community-state dynamics. Reported performance for clinical EHR-based models, particularly tree-based boosting methods, random forest, AutoML, and LSTM, was generally in the low-to-mid 0.80 s for general PTB prediction, whereas transformer-based models achieved AUCs around 0.79. Microbiome-based models demonstrated higher performance for early PTB prediction but more modest performance for overall PTB, while EHG-based DL models reported lower AUCs despite promising signal characteristics, largely reflecting their smaller sample sizes. Collectively, these findings suggest that predictive performance is strongly influenced by dataset characteristics, predictor availability, and methodological design, emphasizing that model performance should be interpreted within the context of each study rather than used to infer the superiority of a particular ML algorithm.

## Discussion

### Data sources and dataset characteristics

Dataset quality emerged as a major determinant of model performance in the reviewed literature. Large administrative databases offered greater statistical power and broader population coverage, whereas prospective cohort studies provided richer clinical detail but were usually constrained by smaller sample sizes. This trade-off between scale and phenotypic granularity was consistent across studies and remains a core challenge in PTB prediction research. Data are central to ML model development because their quality, completeness, and representativeness directly influence prediction performance and clinical applicability [48–50].

The reviewed studies used a wide range of data sources. Several relied on comprehensive EHR datasets that integrated demographic, clinical, laboratory, and imaging information. For example, Zhang et al. [43] and Gao et al. [37] used maternal records containing demographic characteristics, obstetric history, anthropometric measures, physiological parameters, ultrasound findings, and laboratory variables. Other studies used population-level databases. Rocha et al. [36] analyzed national maternal health data, while Ebrahimvandi et al. [38] linked multiple data sources, including the Centers for Disease Control and Prevention (CDC), County Health Rankings, and the Area Health Resources Files, creating a dataset with approximately 380 variables. In contrast, Raja et al. [42] used a public birth registry, and Khan et al. [39] followed 3,509 women prospectively with a smaller but more focused set of 35 features. Although the smaller cohorts were more homogeneous, the large administrative datasets were more heterogeneous and required careful preprocessing, harmonization, and feature selection to reduce noise and bias. Missing data handling also varied across studies: mean imputation [38], multiple imputation using chained equations [47], and complete-case analysis [41]. were all used, each with different implications for bias and data retention.

### Feature importance and clinical relevance

Feature importance methods quantify the contribution of predictors to model output and are particularly useful in PTB research because they improve interpretability and help identify clinically relevant risk factors [51–57]. Across the reviewed studies, previous PTB, cervical length, gestational hypertension, diabetes, preeclampsia, maternal age, BMI, multiple gestation, and ANC attendance were among the most frequently reported predictors. These variables are clinically meaningful because they support different types of action: some identify pregnancies requiring intensified surveillance, whereas others indicate modifiable risk pathways that may benefit from intervention or counseling.

**Fig. 3:**
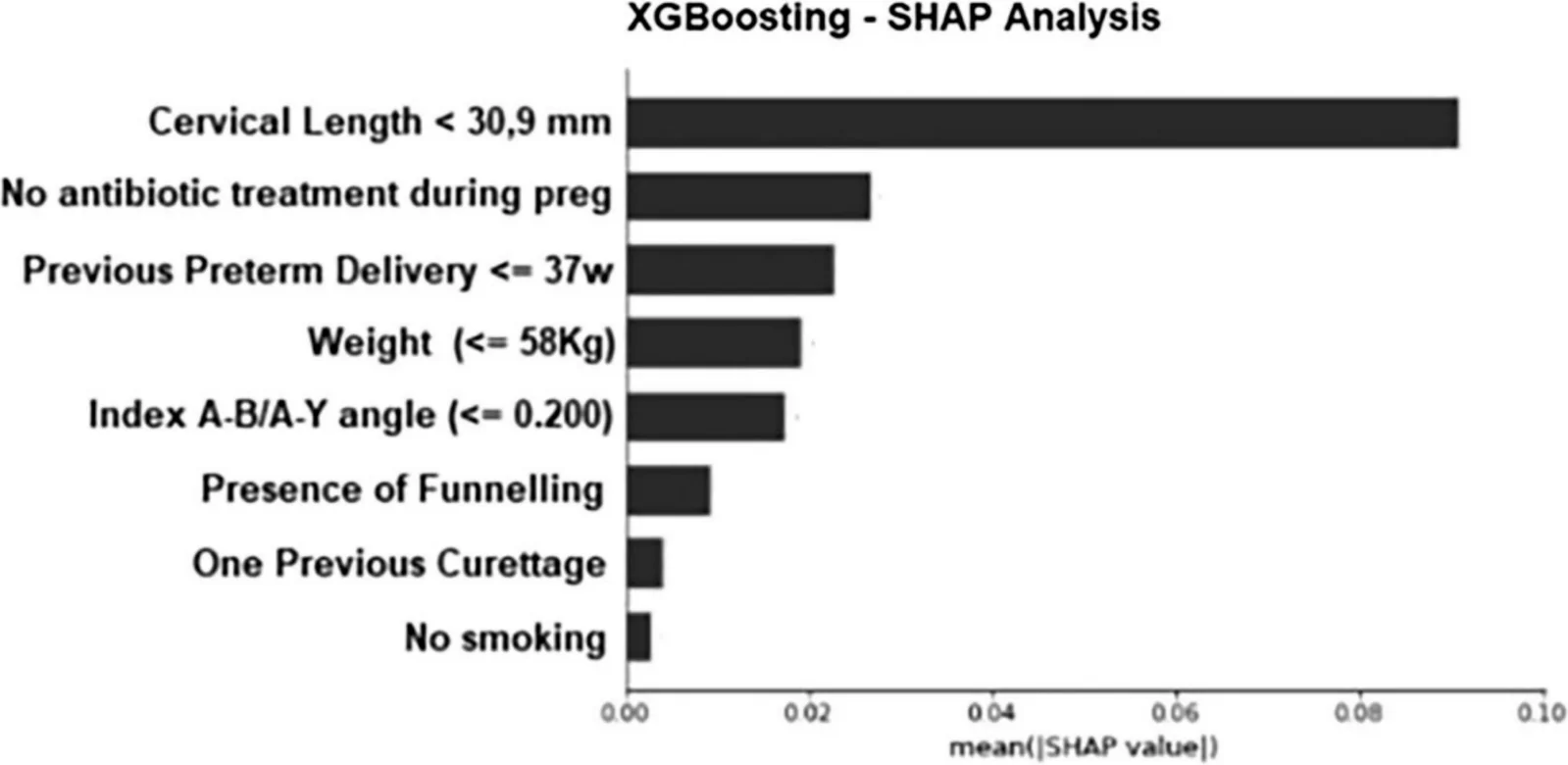
XGBoost—SHAP analysis. Adapted from Andrade Junior et al. [21] with permission under the terms of the CC BY 4.0 License, Copyright 2019

SHAP was used in several studies to explain predictor contributions at the individual level. Ebrahimvandi et al. [38] and Wong et al. [41] reported feature rankings using boosting-based and SHAP-based approaches, respectively. Andrade Junior et al. [21] applied SHAP to identify and interpret the contribution of individual predictors, and the corresponding SHAP feature importance graph is presented in Fig. 3.

**Fig. 4:**
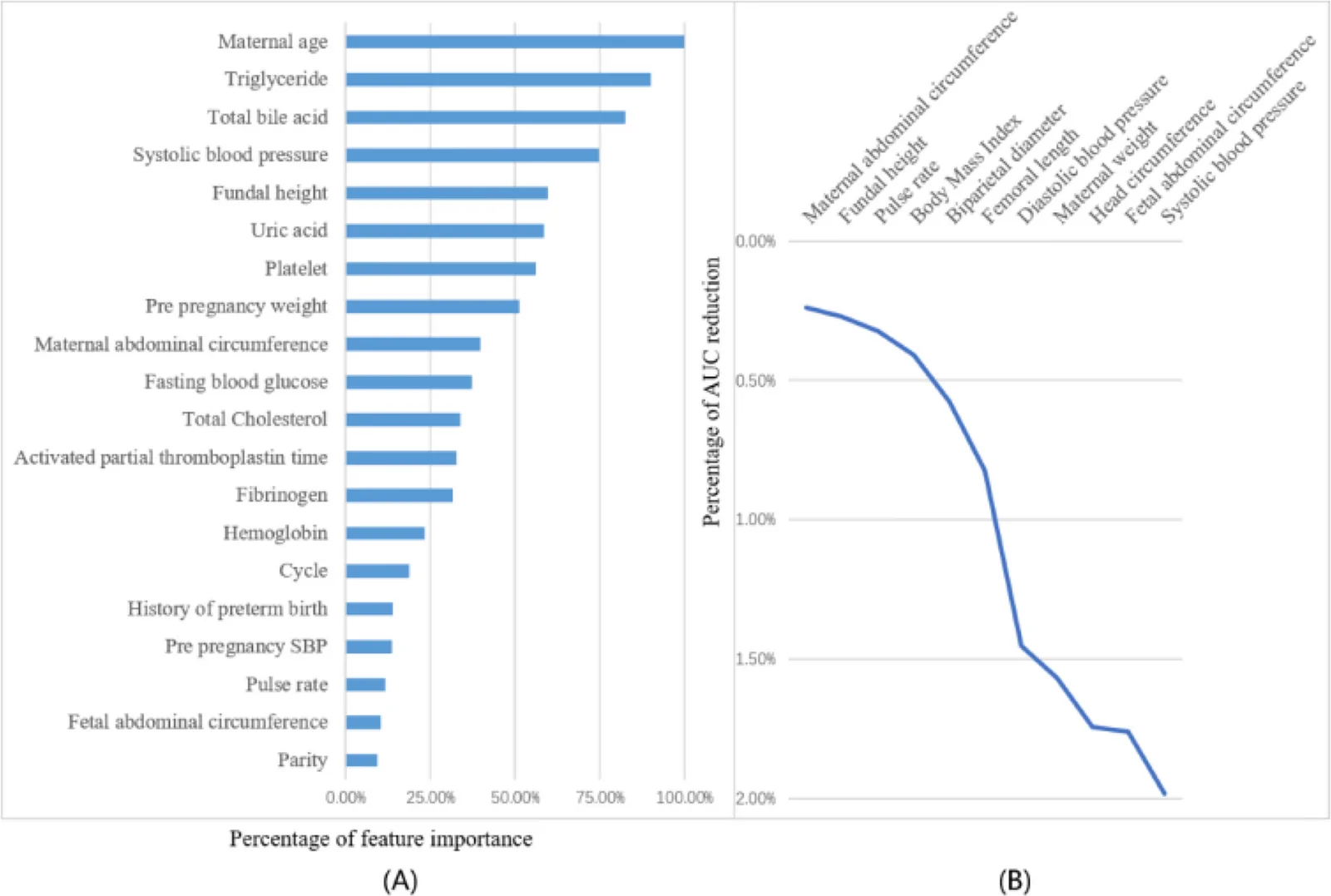
Feature importance graph. Adapted from Zhang et al. [43] with permission under the terms of the CC BY 4.0 License, Copyright 2019

Similarly, Zhang et al. [43] evaluated feature importance in both XGBoost and LSTM models, and the resulting feature importance graph is presented in Fig. 4.

A more clinically useful interpretation is to group predictors into three categories: obstetric history factors such as previous PTB and prior cesarean delivery, current-pregnancy indicators such as short cervical length, hypertensive disorders, threatened miscarriage, and multiple gestation, and maternal background or behavioral factors such as BMI, age, ANC attendance, smoking, and metabolic markers. Taken together, these predictors are repeatedly identified by ML models, but their relative importance varies by population, timing, data availability, and model type; therefore, feature importance should be interpreted as a guide for clinical risk stratification rather than as isolated model output. Although the ranking of predictors varied by study and model class, the recurrence of a small set of biologically plausible variables suggests that PTB prediction is driven by a stable core of obstetric and maternal risk factors. This is clinically important because models are most useful when they identify pathways that can inform management rather than merely produce a risk score. Table 1 shows a clinical interpretation of frequently reported predictors.

**Table 1:** Feature importance summary table

Predictors	Categories	Modifiables	Clinical relevance
Previous PTB	Obstetric history	No	Strong recurrence risk
Cervical length	Biophysical	Partially	Screening target
BMI	Maternal demography	Yes	Preventive intervention
Diabetes	Maternal health	Yes	Management target
Hypertension	Maternal health	Yes	Monitoring target
ANC attendance	Healthcare access	Yes	Intervention opportunity

Although predictor rankings varied across studies and ML algorithms, several variables repeatedly emerged as influential. Previous PTB, cervical length, maternal age, BMI, hypertensive disorders, diabetes, and ANC utilization demonstrated consistent predictive relevance. Importantly, some predictors represent fixed biological risk, whereas others identify opportunities for preventive intervention. This distinction is clinically meaningful because prediction models are most valuable when they identify modifiable pathways that can inform management decisions.

### Evidence synthesis of feature importance

Across the included studies, a consistent pattern emerged regarding the predictors that most strongly contributed to ML models for PTB prediction. Previous PTB, cervical length, maternal age, BMI, hypertensive disorders of pregnancy, diabetes mellitus, and multiple gestation were the most frequently identified predictors, irrespective of the ML algorithm employed. Although the relative importance of individual variables varied across datasets and feature selection techniques, these predictors consistently demonstrated substantial predictive value across geographically and clinically diverse populations. This finding suggests that future ML models should prioritize clinically validated obstetric and maternal health variables before incorporating increasingly complex or high-dimensional feature sets.

Collectively, these findings suggest that successful prediction depends less on identifying novel predictors than on robust integration of well-established clinical risk factors within rigorously validated ML frameworks.

### Comparative model performance and clinical utility

Comparison of performance requires attention to the prediction task and metric used. Studies reporting binary PTB classification commonly used AUC, sensitivity, specificity, and F1-score, whereas studies predicting gestational timing used RMSE or related regression metrics. No single ML algorithm consistently outperformed all others across studies. Performance depended on data quality, cohort composition, prediction window, feature engineering, and validation design. In an early singleton-pregnancy dataset, Andrade Junior et al. [21] reported that a stacking-based ensemble outperformed cervical length alone (AUC 0.808 vs 0.3818). Rocha et al. [36] found that XGBoost achieved the lowest RMSE for delivery-time prediction among six algorithms. Raja et al. [42] reported an accuracy of 0.9 using SVM for PTB prediction. Gao et al. [37] reported an AUC of 0.827 for RNN-based extreme PTB prediction before 28 weeks. Ebrahimvandi et al. [38] found that gradient boosting performed best, with AUC 0.75, sensitivity 0.64, and specificity 0.73. In nulliparous women, Sari et al. [34] showed that ANN performance improved when first- and second-trimester data were combined, reaching an AUC of 0.8. Khan et al. [39]. Khabiti et al. [40] achieved an accuracy of 0.81 and AUC of 0.68 for term birth, spontaneous PTB, and provider-initiated PTB classification, while Wong et al. [41] reported substantial variability across models, with MLP accuracy at 0.57. Zhang et al. [43] found that LSTM achieved an accuracy of 0.739 and an AUC of 0.651. More recent studies have also supported the use of larger datasets and ensemble learning (Kong et al.) Comparative model performance and clinical utility. [45] reported median AUCs of 0.846–0.820 using AutoML on inpatient discharge data. Qian et al. [46] reported AUCs of 0.826 for random forest and 0.851 for LSTM with external validation, and Liu et al. [47] reported AUCs of 0.701–0.704 with good calibration and decision-curve benefit in a large NVSS cohort.

Taken together, these findings show that model performance varies substantially by cohort and task, and that high discrimination alone does not guarantee clinical usefulness. In practice, the most informative models were those trained on early pregnancy data, longitudinal records, or subgroup-specific cohorts. For this reason, model selection should be driven by clinical context rather than algorithmic popularity. A general overview of some of the studies is shown in Table 2 below.

**Table 2:** Summary of evaluation of representative studies involving ML for PTB prediction. LR, logistic regression; SVM, support vector machine; DT, decision tree; RF, random forest; ANN, artificial neural network; RNN, recurrent neural network; GBM, gradient boosting machine; LGBM, light gradient boosting; XGB, extreme gradient boosting; LSTM, long short-term memory; MLP, multilayer perceptron; EN, elastic net; LASSO, Least Absolute Shrinkage and Selection Operator; SBELM, stacking-based ensemble learning; MR-PB-PFS, map reduce-based ML

Ref	Data type	Sample size	Method	Performance	Top factors
Andrade Júnior et al. [21]	Numeric	524	SBELM, ANN, LR	AUC: 0.318—0.808	Short cervical length, PTB history, and no antibiotic treatment during pregnancy
Gao et al. [58]	Numeric	25,689	RNN	AUC: 0.827	Multiple gestation, Short cervical length, Maternal health complications
Ebrahimvandi et al. [38]	Numeric	3,610,827	LR-EN, RF, GB, LGBM	AUC: 0.661- 0.7591	Gestational hypertension, Interval between births, and history of PTB
Raja et al. [43]	Numeric	112,963	ANN, LR	AUC: 0.6—0.8	Having diabetic mellitus, a history of abortion, and Pregnancy abnormalities
Khan et al. [40]	Numeric	3509	XGB, SVM, LR, MLP, GBM, RF	AUC: 0.720—0.735	Previous PTB history, Previous cesarean section, and Preeclampsia during pregnancy. For parous and BMI, age, and amniotic infection in nulliparous women
Khatibi et al. [41]	Numeric	1,400,000	MR-PB-PFS, SBELM	AUC: 0.68 and accuracy: 0.81	Gestational diabetes, Maternal illnesses, Chronic hypertension
Wong et al. [42]	Numeric	958,729	DT, RF, XGB, MLP	Accuracy: 0.98—0.9147 and AUC: 0.57—0.86	Threatened miscarriage, PTB history, and Multiple gestations
Pons-Duran et al. [59]	Numeric	2493	XGB	AUC: 0.60	-
Zhang et al. [60]	Numeric	5187	RNN, LSTM	Accuracy: 0.739 and AUC: 0.651	Hypertension, Diabetes, Body Lipid
Abraham et al. [45]	Numeric	35,282	XGB	Precision: 0.96, Recall: 0.96, and AUC: 0.44	Hypertension

Collectively, large variation in reported performance reflected methodological heterogeneity across studies, emphasizing that direct comparison of discrimination metrics should be interpreted cautiously.

### Evidence synthesis and practical recommendations

Across the reviewed studies, several consistent patterns emerged despite methodological heterogeneity. First, models developed using longitudinal EHR data generally achieved more robust predictive performance than those relying solely on cross-sectional clinical variables, suggesting that temporal information captures progressive physiological changes preceding PTB.

Second, ensemble learning approaches, particularly gradient boosting, random forests, and stacking models, consistently demonstrated strong discrimination across diverse populations, although no single algorithm was universally superior. Instead, model performance depended primarily on data quality, predictor availability, and validation strategy rather than algorithm choice alone.

A third recurring finding was the stability of a relatively small group of clinically meaningful predictors, including previous PTB, cervical length, maternal age, hypertensive disorders, diabetes, BMI, and ANC utilization. Although their relative importance differed among studies, these predictors repeatedly contributed to successful risk prediction, indicating that future models should prioritize clinically validated variables before incorporating increasingly complex feature sets.

### Prediction timing and clinical actionability

Prediction timing is a critical determinant of clinical utility. Models that identify risk in the first trimester or early second trimester provide greater opportunity for preventive intervention, enhanced surveillance, and counseling. Later-pregnancy predictions may achieve acceptable statistical performance but offer less time for action. This pattern was evident in studies showing better performance when trimester data were combined [38] and in sequence-based models that used early pregnancy to 28 weeks of gestation [43].

### Critical appraisal of evidence quality

Many studies used retrospective designs, single-center cohorts, or internal validation only, which may inflate apparent performance and limit generalizability. In addition, inconsistent missing-data handling and limited calibration reporting make it difficult to judge the reliability of predicted probabilities [38, 41, 47, 61]. Studies with stronger methodological credibility generally combined clinically relevant predictors, transparent preprocessing, and at least some form of external or temporally separated validation. Overall, the evidence base supports the promise of ML for PTB prediction, but more rigorous validation and reporting are still needed before routine clinical translation (Table 3).

**Table 3:** Evidence quality assessment

Domains	Common Issues
Missing data	Inconsistent handling
Validation	Internal only
Calibration	Rarely reported
Generalizability	Limited
Bias	Selection bias

### Clinical implementation of ML models for PTB prediction

Although many ML models demonstrated promising predictive performance, successful translation into routine obstetric practice requires integration within established clinical workflows rather than use as standalone prediction tools. In practice, ML models are most likely to function as clinical decision support systems integrated within EHR platforms, where routinely collected maternal demographic characteristics, obstetric history, laboratory investigations, and ANC data can be processed automatically to generate individualized risk estimates during early pregnancy or subsequent antenatal visits.

Rather than replacing clinical judgment, these models should complement existing risk assessment by identifying women who may benefit from closer surveillance or evidence-based preventive interventions. For example, women identified as high risk may undergo additional cervical length assessment, receive progesterone prophylaxis when clinically indicate, undergo more frequent antenatal monitoring, or be referred to specialist maternal–fetal medicine services according to established clinical guidelines. Conversely, women classified as low risk may continue routine ANC, thereby supporting more efficient allocation of healthcare resources.

Importantly, implementation should only occur following rigorous external validation, calibration assessment, prospective evaluation, and demonstration of clinical benefit across diverse healthcare settings. Transparent and explainable prediction models capable of providing interpretable explanations for individual predictions are likely to improve clinician confidence, facilitate shared decision-making, and promote responsible integration into routine obstetric care.

Collectively, these findings indicate that improvements in predictive accuracy alone are unlikely to facilitate clinical adoption. Successful implementation will require integration into routine antenatal workflows, rigorous prospective validation, transparent model interpretation, and demonstration that ML-assisted decision support improves maternal and neonatal outcomes beyond existing clinical practice.

### Interpretation of variation in predictive performance

The huge variation in reported predictive performance across studies should not be interpreted as evidence that one ML algorithm is consistently superior to another. Instead, performance differences are more likely to reflect heterogeneity in study populations, sample sizes, outcome definitions, prediction windows, predictor availability, feature engineering strategies, handling of missing data, class imbalance, validation procedures, and outcome prevalence. Studies using larger multicenter datasets, clinically relevant predictors, and rigorous external validation generally provide more reliable estimates of model performance than studies based on smaller, single-center cohorts evaluated solely through internal validation. Consequently, reported discrimination metrics should be interpreted within the methodological context of each study rather than compared directly across investigations.

## Limitations of current evidence

Several methodological limitations recur across the reviewed literature and restrict the clinical translation of ML models for PTB prediction. Most studies used retrospective designs, single-center cohorts, or internal validation only, which may inflate apparent performance and limit generalizability. Data quality was also variable, with incomplete records, non-standardized definitions, and inconsistent preprocessing strategies across studies. In addition, many datasets included features that were not clearly PTB-specific, which may introduce noise and reduce model robustness [16]. The strongest limitations were the limited use of external validation, calibration assessment, real-world deployment testing, and clinical impact evaluation. Few studies validated their models on independent datasets, and even fewer examined whether predicted probabilities were well calibrated. This is important because a model that ranks risk well may still produce unreliable absolute risk estimates. Similarly, little evidence exists on whether these models improve clinical decisions, workflow efficiency, or maternal and neonatal outcomes in practice. These gaps make it difficult to judge whether the reported performance can be reproduced in other settings or translated into routine obstetric care [60, 62].

Feature selection and interpretability remain additional concerns. Although some studies identified clinically plausible predictors, many high-performing models, particularly boosting-based and neural network approaches, function as “black boxes,” making their outputs difficult to interpret in clinical settings. This limits acceptance by clinicians and weakens the usefulness of model outputs for counseling and decision support [58, 59]. Finally, many studies did not clearly distinguish modifiable from non-modifiable predictors, which reduces the practical value of the reported risk factors for prevention-oriented care. Future PTB prediction studies should therefore prioritize external validation, calibration reporting, transparent preprocessing, subgroup analysis, and evaluation of clinical impact alongside discrimination metrics (Table 4).

**Table 4:** Key challenges and implications

Domains	Common issues	Implications
Data quality	Incomplete or non-standardized data	Reduced robustness and comparability
Validation	Internal validation only	Inflated performance estimates
Calibration	Rarely reported	Uncertain probability reliability
Deployment	Rarely tested in practice	Limited translational value
Clinical impact	Not assessed	Unclear effect on outcomes
Bias	Selection bias and missing-data handling	Reduced generalizability

### Quality of evidence and methodological limitations

Although the reviewed studies collectively demonstrate the promise of ML for PTB prediction, the overall strength of the evidence should be interpreted cautiously because several methodological limitations were consistently observed. Most studies employed retrospective observational datasets, many relied on single-center populations, and relatively few performed external validation. Consequently, the reported predictive performance may overestimate real-world effectiveness when models are applied to different clinical settings or populations. Furthermore, inconsistent definitions of PTB, variation in predictor selection, heterogeneous feature engineering approaches, and differences in validation strategies reduce the comparability of published models. These methodological differences limit direct comparison of reported performance metrics and emphasize that predictive accuracy should not be interpreted independently of study design and validation quality.

## Future directions

Future research should prioritize prospective, multicenter studies using standardized definitions of predictors and outcomes to improve model generalizability and comparability. Model evaluation should routinely include external validation, calibration, and explainability to support clinical interpretation and implementation. In addition, prospective implementation studies are needed to assess integration into routine ANC and EHR systems, as well as clinician acceptance, cost-effectiveness, equity across diverse populations, and the impact of ML-assisted risk prediction on maternal and neonatal outcomes.

### Data quality and standardization

Future progress will depend heavily on the availability of high-quality, standardized, and representative datasets. The reviewed studies demonstrated substantial heterogeneity in data sources, predictor definitions, preprocessing procedures, and outcome classifications, which limits comparability across studies and may contribute to variability in reported performance [63]. Greater harmonization of data collection practices, together with the inclusion of clinically relevant demographic, obstetric, environmental, and socioeconomic variables, could improve model robustness and generalizability. Collaborative multicenter initiatives and data-sharing frameworks may further facilitate the development of more representative datasets and support external validation across diverse populations [64].

### Explainability and clinical interpretability

Model interpretability remains a major barrier to clinical adoption. Although complex ML algorithms often achieve strong predictive performance, clinicians must be able to understand and trust model outputs before incorporating them into patient care [65]. Future research should therefore prioritize explainable AI approaches that provide transparent and clinically meaningful explanations for predictions. Methods such as SHAP and other feature-attribution techniques can help identify the factors driving risk estimates and improve clinician confidence in model-assisted decision-making [66, 67]. Emphasis should be placed on developing models that balance predictive accuracy with interpretability rather than optimizing performance alone.

### Prospective validation and clinical implementation

The most important gap identified in this review is the limited evaluation of models beyond retrospective datasets. Many studies relied on internal validation, while relatively few conducted external validations or assessed performance in real-world clinical settings. Future research should therefore prioritize prospective, multicenter validation studies that evaluate model performance across different healthcare systems and patient populations. Additional metrics, such as positive predictive value (PPV), F1-score, and precision-recall curves, should assess calibration, clinical utility, workflow integration, and impact on maternal and neonatal outcomes. Ultimately, PTB prediction models should function as clinical decision-support tools that facilitate early intervention, targeted surveillance, and individualized risk management rather than as stand-alone predictive algorithms [64, 68–70].

A summary of practical recommendations from reviewed evidence is listed below in Table 5.

**Table 5:** Practical recommendations derived from the reviewed evidence

Evidence gap	Recommendation	Expected impact
Small single-center datasets	Multicenter prospective cohorts	Better generalizability
Internal validation only	External validation	Reliable performance
Poor calibration	Report calibration	Safer risk estimates
Black-box models	Explainable AI	Clinical trust
Heterogeneous reporting	TRIPOD-AI reporting	Reproducibility
Lack of deployment	Prospective implementation studies	Clinical adoption

## Conclusion

ML has emerged as a promising approach for improving the prediction of PTB, a leading cause of neonatal morbidity and mortality worldwide. This review demonstrates that ML models can leverage diverse clinical, demographic, laboratory, and pregnancy-related data to identify women at increased risk of PTB and potentially support earlier and more targeted preventive interventions. Across the reviewed studies, maternal medical history, demographic characteristics, obstetric factors, and longitudinal pregnancy data were among the most consistently reported predictors, highlighting their importance in PTB risk assessment.

Despite encouraging results, substantial challenges remain. The reviewed studies exhibited considerable heterogeneity in data sources, cohort characteristics, outcome definitions, feature selection methods, model development strategies, validation approaches, and performance reporting. Furthermore, limited external validation, inadequate calibration assessment, and variability in model interpretability restrict the generalizability and clinical applicability of many proposed models. These methodological limitations continue to hinder the translation of ML-based PTB prediction tools into routine obstetric practice.

Future research should prioritize the development of robust, transparent, and clinically interpretable models using large, diverse, and representative datasets. Greater emphasis should be placed on standardized reporting, external validation across different populations and healthcare settings, calibration assessment, and prospective clinical evaluation. In addition, the integration of explainable artificial intelligence techniques may enhance clinician trust and facilitate the adoption of ML-based decision-support systems in maternal healthcare.

The evidence synthesized in this review suggests that the future success of ML for PTB prediction will depend less on developing increasingly complex algorithms and more on improving data quality, methodological rigor, external validation, explainability, and clinical implementation. Future research should therefore prioritize reproducible, transparent, and clinically integrated prediction models capable of supporting real-time obstetric decision-making rather than focusing solely on incremental gains in predictive accuracy.

Although ML models demonstrate encouraging predictive performance, their ultimate clinical value will depend on successful integration into routine obstetric workflows. Future research should therefore prioritize prospective implementation, explainability, external validation, and demonstration of clinical effectiveness to ensure that prediction models translate into meaningful improvements in maternal and neonatal outcomes.

## Data Availability

No datasets were generated or analysed during the current study.

## Abbreviations

AI: Artificial Intelligence
ANC: Antenatal Care
ANN: Artificial Neural Network
AUC: Area Under the Receiver Operating Characteristic Curve
AutoML: Automated Machine Learning
BMI: Body Mass Index
CDC: Centers for Disease Control and Prevention
DL: Deep Learning
DT: Decision Tree
EHG: Electrohysterogram
HER: Electronic Health Record
EMR: Electronic Medical Record
EN: Elastic Net
GB: Gradient Boosting
GBM: Gradient Boosting Machine
LASSO: Least Absolute Shrinkage and Selection Operator
LGBM: Light Gradient Boosting Machine
LR: Logistic Regression
LSTM: Long Short-Term Memory
ML: Machine Learning
MLP: Multilayer Perceptron
MR-PB-PFS: Map Reduce-Based Preterm Birth Prediction Feature Selection
NPV: Negative Predictive Value
NVSS: National Vital Statistics System
PPV: Positive Predictive Value
PROM: Premature Rupture of Membranes
PTB: Preterm Birth
RF: Random Forest
RMSE: Root Mean Square Error
RNN: Recurrent Neural Network
SHAP: Shapley Additive Explanations
SBELM: Stacking-Based Ensemble Learning
SVM: Support Vector Machine
WHO: World Health Organization
XGB: Extreme Gradient Boosting
